# Immune landscape and prognostic immune-related signature in KRAS-mutated lung adenocarcinoma

**DOI:** 10.18632/aging.204770

**Published:** 2023-06-06

**Authors:** Xinyi Peng, Zhenqi Xia, Yong Guo, Yan Li

**Affiliations:** 1 Department of Oncology, Wenzhou Hospital of Traditional Chinese Medicine Affiliated to Zhejiang Chinese Medicine University, Wenzhou, Zhejiang 325000, China; 2 The First Clinical College of Zhejiang Chinese Medical University, Hangzhou, Zhejiang 310000, China; 3 Department of Oncology, Zhejiang Provincial Hospital of Traditional Chinese Medicine, Hangzhou, Zhejiang 310000, China

**Keywords:** lung adenocarcinoma, KRAS, immune landscape, risk signature, immunotherapy responses

## Abstract

The heterogeneity of lung adenocarcinoma (LUAD) indicated that target therapies and immunotherapies may not be effective in all patients. The exploration of the feature of the immune landscape of different gene mutations may provide novel perspectives. In this study, we obtained LUAD samples from The Cancer Genome Atlas. By applying ESTIMATE and ssGSEA, KRAS-mutated group was discovered to be associated with lower immune infiltration, lower expression of immune checkpoints, especially, a lower abundance of B cell, CD8+ T cell, dendritic cell, natural killer cell, and macrophage, higher abundance of neutrophil and endothelial cell. Through ssGSEA, we found that the process of antigen-presenting cell co-inhibition and co-stimulation were inhibited, cytolytic activity and human leukocyte antigen molecules were downregulated in the KRAS-mutated group. And KRAS mutation is negatively related to antigen presentation and procession, cytotoxic lymphocyte activity, cytolytic activities, and cytokine interaction signaling pathway via gene function enrichment analysis. Finally, 24 immune-related genes were identified to establish an immune-related gene signature with excellent prognostic prediction capacity, whose 1-, 3- and 5-year AUCs were 0.893, 0.986, and 0.999. Our findings elucidate the features of the immune landscape of KRAS-mutated groups and successfully established a prognostic signature on the basis of immune-related genes in LUAD.

## INTRODUCTION

Lung cancer remains a severe challenge to human health with the second highest morbidity and the first highest mortality worldwide [1]. Histologically, lung cancer could be assigned into non-small-cell lung cancer (NSCLC) and small-cell lung cancer (SCLC). And NSCLC approximately accounts for 80% of lung cancer. As lung adenocarcinoma (LUAD) makes up above 50% of NSCLC, it is a very common pathological type clinically. The treatment of LUAD has always been the focus of research in the field of lung cancer [2]. Mutations of critical genes could impact the proliferation, metabolism, apoptosis, and invasion of LUAD, including TP53, EGFR, ROS1, KRAS, NTRK and so on [3]. Despite recent developments in immunotherapy (Immune checkpoint inhibitors), molecular characterization, and targeted therapy, the 5-year overall survival (OS) in LUAD remains poor [4]. And biomarkers that assess the prognosis and immunotherapy sensitivity of LUAD are still unsatisfactory.

Kirsten Rat sarcoma virus (KRAS) is a member of RAS gene family, encoding small GTPases [5]. KRAS mutations are highly frequent oncogene in cancer, up to 20~25% of LUAD [6, 7]. It has been reported that, compared with KRAS-wild type, LUAD with KRAS mutation was related to a worse prognosis, especially in the advanced-stage subgroup [8, 9].

Although KRAS oncogene was used to be considered an “undruggable” target, in recent years, some targeted drugs targeting KRAS p.G12C mutation in LUAD had gradually entered the phase of clinical trials, for instance, AMG510 and Adagrasib [10, 11]. This means researchers have achieved a historical breakthrough in developing effective KRAS G12C inhibitors, and more drugs targeting other KRAS-mutated isoforms will be available in the future [12].

Whether KRAS could be used as a biomarker for immunotherapy remains uncertain. Some studies suggested that KRAS mutation cannot be used as a biomarker of immunotherapy. Passiglia et al. [13] confirmed that there was no significant difference in objective response rate (ORR) and OS of NSCLC patients with KRAS mutation who received nivolumab. However, Coelho et al. [14] found that KRAS can promote programmed cell death-Ligand 1 (PD-L1) stability to increase PD-L1 expression, through modulating the AU-rich element-binding protein. According to previous research, KRAS mutation participates in the formation of tumor immunosuppressive microenvironment and may inhibit immunotherapy response. Gao et al. found that KRAS G12D/TP53 co-mutation activated immune suppression, which was a potential negatively predictive biomarker of immunotherapies for LUAD patients [15]. Pinto et al. discovered patients with KRAS mutation have low B cell infiltration, which may be the reason for their immune suppression [10].

The current studies on the immune microenvironment of KRAS mutant patients are scattered and do not fully reflect the characteristics of the immune landscape of KRAS-mutated LUAD. Hence, based on transcriptome data of LUAD from the public database, this study utilized bioinformatic technology to comprehensively analyze the immune landscape characteristics of KRAS-mutated LUAD from various aspects, explored the underlying immune mechanism, established corresponding immune-related prognostic signatures, and identified hub genes.

## MATERIALS AND METHODS

### Data acquisition

**Figure 1:**
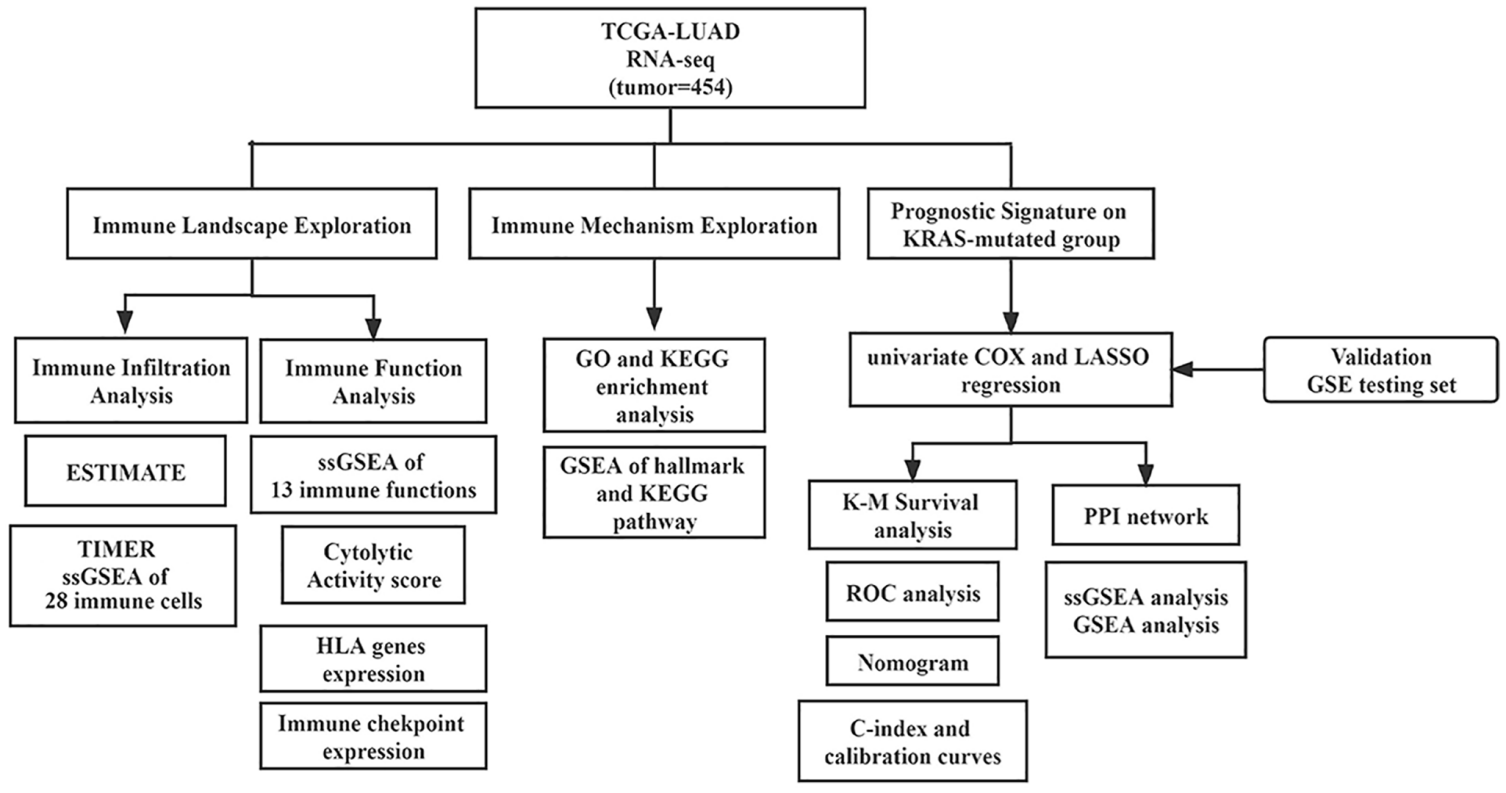
Flowchart of this study.

We downloaded high-throughput RNA sequencing (TPM), corresponding clinicopathological data, and somatic mutation information of LUAD from The Cancer Genome Atlas (TCGA) on July 28, 2022. TCGA database has been updated with TPM data. And GSE72094 dataset was acquired from Gene Expression Omnibus (GEO), serving as an independent validation cohort. Mutation Annotation Format (MAF) files of LUAD were merged to obtain somatic mutation data. Only cases with primary lung adenocarcinoma, complete follow-up data, and clear KRAS status were included for subsequent analysis. Genes with no expression in more than half samples were excluded. And TPM data were normalized by log2(TPM+1). We used “removeBatchEffect” method from “limma” package to remove batch effects. The 1793 immune-related genes (IRGs) were collected from the immPort database (“https://www.immport.org/resources”). The work flowchart of our study is displayed in Figure 1.

### The exploration of the landscapes in KRAS-driven lung adenocarcinoma

We first utilized the ESTIMATE algorithm to evaluate the stromal score, immune score, and tumor purity of different KRAS status subgroups via the “estimate” package. Secondly, to analyze the immune infiltration of the KRAS-mutated and KRAS-wild groups in more detail, TIMER (http://timer.cistrome.org/) and single sample Gene Set Enrichment Analysis (ssGSEA) algorithm were employed to calculate the abundances of immune cells in LUAD. Meanwhile, we also compared the expressions of human leukocyte antigen (HLA)-related genes and immune checkpoints under different KRAS statuses. The “GSVA” and “GSEABase” packages were utilized in this process.

### Biological function analysis

We conducted differential expression analysis between the KRAS-mutated and KRAS-wild subgroups by “limma” package. The filtering conditions for differentially expressed genes (DEGs) were log2|Fold Change| > 0.58 and adjust *p* value < 0.05. The result was prepared for enrichment analysis, containing Kyoto Encyclopedia of Genes and Genomes (KEGG) and Gene Ontology (GO) sets. And the outcomes would be visualized via the “ggplot 2” and “goplot” packages. In addition, we also utilized Gene set enrichment analysis (GSEA) to probe activating and suppressing signaling pathways. The gene sets were acquired from the MsigDB (http://www.gsea-msigdb.org/gsea/msigdb/).

### Construction and validation of the immune-related signature

Since we found that different KRAS states have different immune microenvironments, we decided to establish immune-related prognostic signatures on KRAS mutated group. TCGA cohort served as the training set, and GSE72094 was the testing set. We firstly applied the univariate COX analysis to screen out the IRGs which were strongly correlated with the survival of patients in the KRAS-mutated subgroup of the TCGA cohort. These prognosis-related IRGs were then put into the least absolute shrinkage and selection operator (LASSO) analysis to eliminate the over-fitting genes via “glmnet” package. The risk score is determined using the following formula:


$$risk\;score=\sum_{k=\text{1}}^{n}\text{(}Coef\;\text{(}RNAk\text{)}\times exp\;\text{(}RNAk\text{))}$$

Exp (RNA) represented the expression value of the selected IRGs, and coef (RNA) is the corresponding risk coefficient of the selected IRGs. And samples were assigned to different risk subgroups based on the median value.

For evaluating the prognostic prediction capacity of this signature, we conducted Kaplan-Meier analysis, time-dependent receiver operating characteristic (timeROC), and calculated the area under the curve (AUC). The “survival”, “survminer” and “timeROC” packages were utilized in this progress.

### Independence detection of the signature and establishment of the prognostic nomogram

Univariate and multivariate Cox regression analyses were conducted to screen the independent prognostic factors by “survival” package. Then, a nomogram was constructed. Meanwhile, the Concordance index (C-index) was calculated and calibration curves were drawn to investigate the 1-, 3- and 5-year OS probabilities. The “rms” package was used in this process.

### PPI network and identification of hub genes

After identifying signature genes in KRAS-mutated subgroups, Protein-Protein Interaction (PPI) network (minimum required interaction score was 0.40) was generated using the STRING database (https://cn.string-db.org/). The top 5 hub genes were determined by the Cytoscape software plugin cytoHubba (3.10.0 version).

### Functional enrichment analysis

Spearman correlation analysis was performed to detect the pathways correlated with the risk score, using the risk score of samples and the ssGSEA activity score of pathways of hallmark and KEGG (*p* < 0.05). And the top 5 activated and suppressed pathways of KEGG in the high-risk subgroup selected by the GSEA algorithm were visualized using the GSEA plot. The “clusterProfiler” and “enrichplot” packages were employed in this process. The pathways of KEGG and hallmarks were acquired from the MsigDB.

### Statistical analysis

All statistical calculation were accomplished in R software (version 4.1.2). The comparison between two groups of the normal distributed quantitative data was implemented through Student’s *t*-test, while the non-normal data was implemented by the Mann-Whitney *U* test. And two groups of qualitative data were compared by Chi-square test and Fisher’s test.

## RESULTS

### Data collection

By using the criteria, 454 samples from TCGA and 359 samples from GEO were used for the analysis. The characteristic feature of the TCGA cohort and GSE72094 cohort were displayed in Table 1.

**Table 1:** Characteristics of patients in the TCGA-LUAD and GSE72094.

	**TCGA cohort (*n* = 454)**	**GSE72094 set (*n* = 398)**	***P*-value**
**Age**
<65	195 (43.0%)	118 (29.6%)	<0.05
≥65	238 (52.4%)	280 (70.4%)	
Unknow	21 (4.6%)	0	
**Gender**
Female	250 (55.1%)	222 (55.8%)	>0.05
Male	204 (44.9%)	176 (44.2%)	
**KRAS status**
Mutated	121 (26.7%)	139 (34.9%)	
Wild	333 (73.3%)	259 (65.1%)	
**Pathologic stage**
Stage I	247 (54.4%)	254 (63.8%)	>0.05
Stage II	105 (23.1%)	67 (16.8%)	
Stage III	72 (15.9%)	57 (14.3%)	
Stage IV	23 (5.1%)	15 (3.8%)	
Unknow	7 (1.5%)	5 (1.3%)	
**T stage**
T1	154 (33.9%)	−	
T2	243 (53.5%)	−	
T3	37 (8.1%)	−	
T4	18 (4.0%)	−	
Unknow	2 (0.4%)	−	
**N stage**
N0	295 (65.0%)	−	
N1	85 (18.7%)	−	
N2	62 (13.7%)	−	
N3	2 (0.4%)	−	
Unknow	10 (2.2%)	−	
**M stage**
M0	427 (94.1%)	−	
M1	23 (5.1%)	−	
Unknow	4 (0.9%)	−	

**Figure 2:**
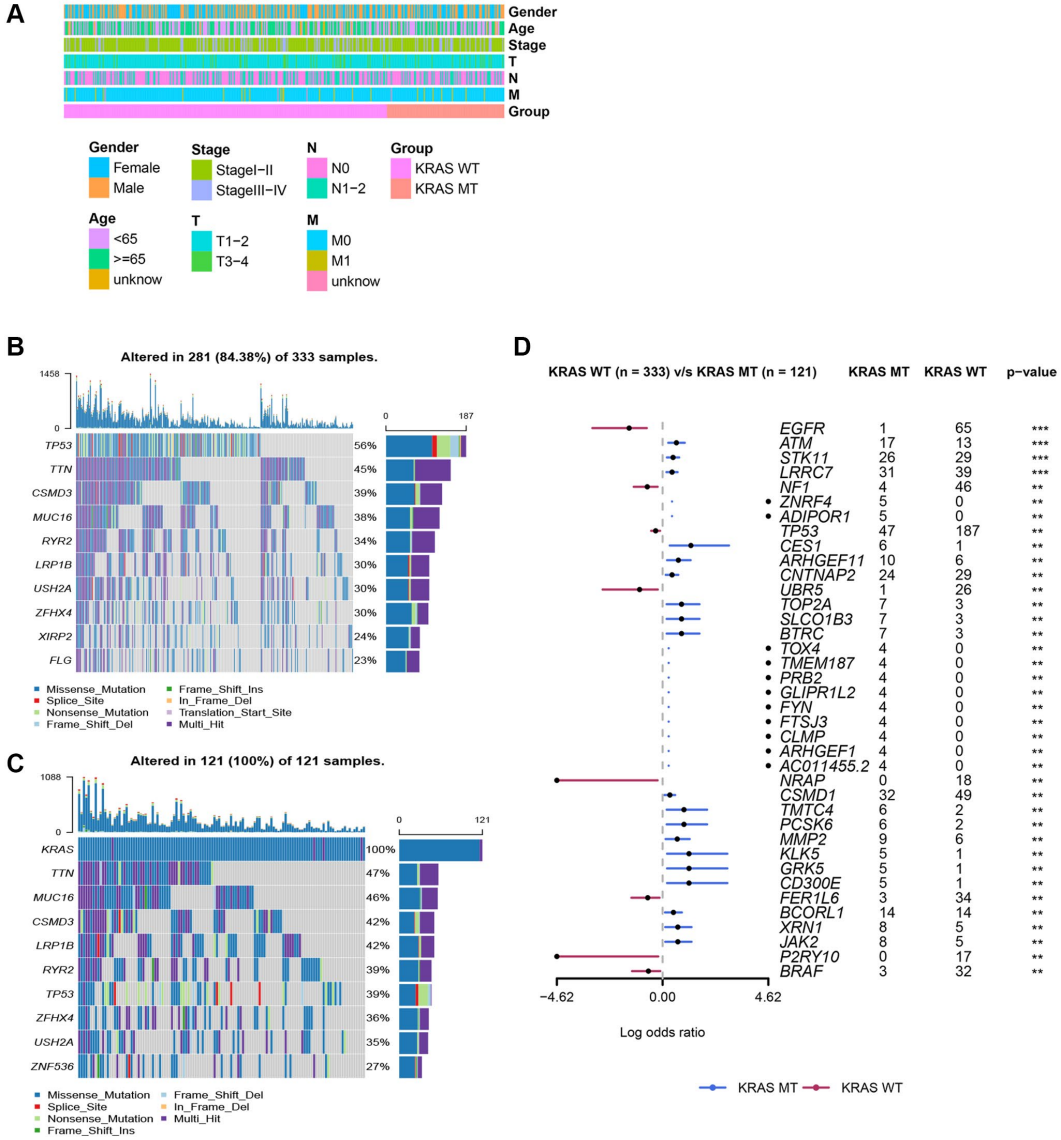
**Clinicopathologic features of LUAD and somatic mutation in KRAS-mutated and KRAS-wild groups.** (**A**) The comparison of clinicopathologic features between KRAS-mutated and KRAS-wild groups. (**B**) The waterfall plot of top 10 mutation genes in KRAS-wild group. (**C**) The waterfall plot of top 10 mutation genes in KRAS-mutated group. (**D**) The forest plot of genes with different mutation frequencies between the KRAS-mutated and KRAS-wild groups. (^*^p < 0.05, ^**^p < 0.01, ^***^p < 0.001, ^****^p < 0.0001).

The TCGA cohort contained 121 KRAS-mutated samples and 333 KRAS-wild samples. The comparison of vital clinical information showed, there were no significant difference in gender, age, stage, T, N, and M between KRAS-mutated and KRAS-wild type groups (Figure 2A). And based on “maf tool” package, the oncoplots displayed the top 10 mutant genes in the groups with different KRAS statuses (Figure 2B, 2C). Genes with different mutation frequencies between two groups are visualized in Figure 2D. The mutation frequencies of EGFR, NF1, and TP53, are lower than that in the KRAS-wild group. And the remaining genes in the forest plot were mutated more frequently in the KRAS-mutated group than in the KRAS-wild group, especially ATM, STK11, LRRC7, CES1, ARHGEF11, CNTNAP2, TOP2A, SLCO1B3, and BTRC.

### The immune landscape of KRAS-driven lung adenocarcinoma

**Figure 3:**
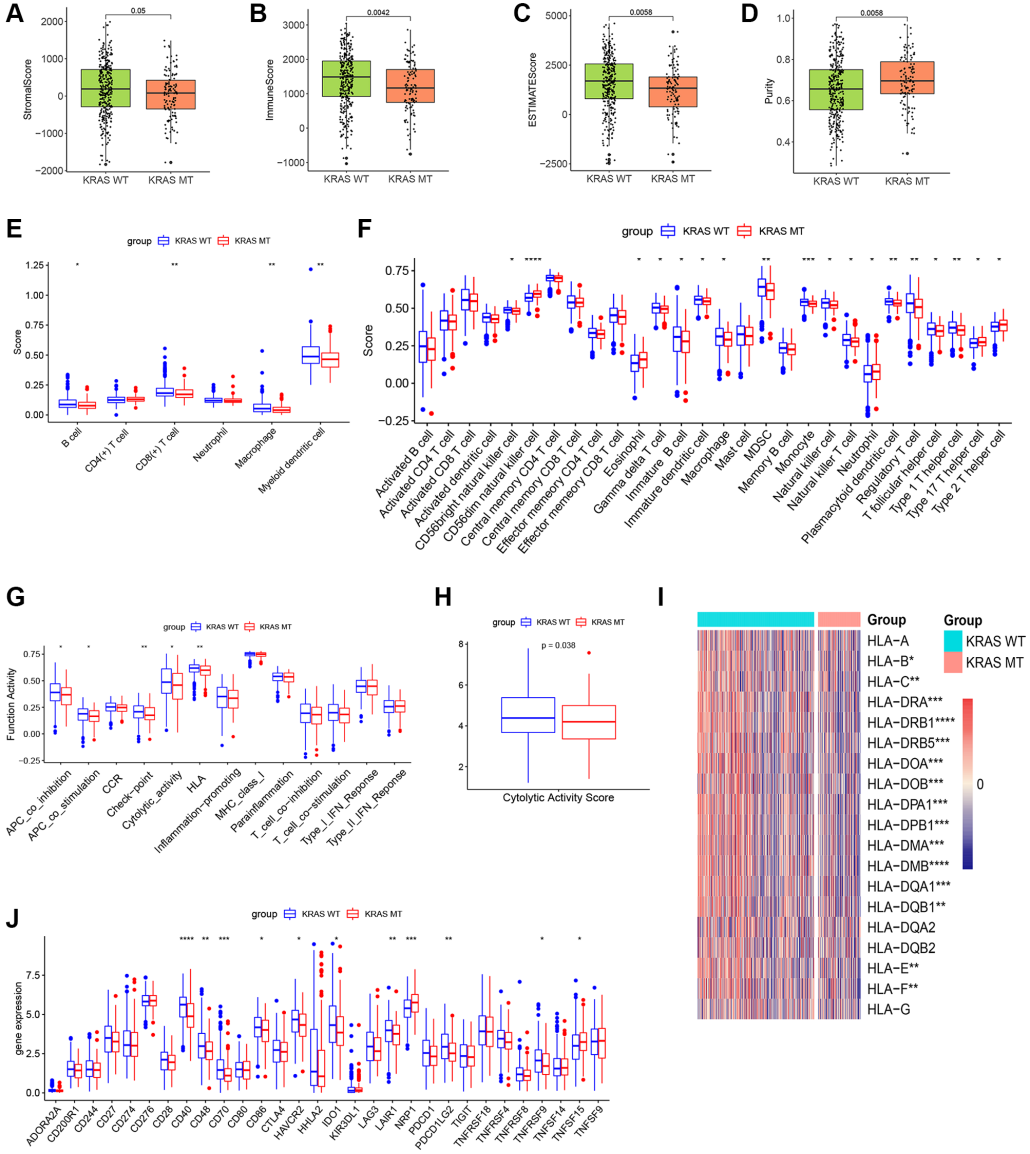
**The plots of immune landscape KRAS-mutated and KRAS-wild groups.** (**A**–**D**) Boxplots of the stromal score, immuno score, ESTIMATE score, and purity score in the 2 groups. (**E**) Boxplot of TIMER score in the 2 groups. (**F**) Boxplot of ssGSEA score of 28 immune cells in the 2 groups. (**G**) Boxplot of ssGSEA score of 13 immune functions in the 2 groups. (**H**) Boxplot of cytolytic activity score in the 2 groups. (**I**) Heatmap of expression values of HLA molecules in the 2 groups. (**J**) Boxplot of expression values of immune checkpoints in the 2 groups. (^*^p < 0.05, ^**^p < 0.01, ^***^p < 0.001, ^****^p < 0.0001).

The result of ESTIMATE revealed the stromal score, immune score, and ESTIMATE score were inferior, while the purity was higher in the KRAS-mutated group, suggesting a lower overall immune level and immunogenicity of the tumor microenvironment in the KRAS-mutated group (Figure 3A–3D). The result of TIMER further elucidated the abundance of B cells, CD8+ T cells, macrophages, and myeloid dendritic cells in the KRAS-mutated group were lower (Figure 3E). The result of ssGSEA of 28 immune cells suggested the abundance of immature B cell, immature dendritic cell macrophage, MDSC, monocyte, natural killer cell, CD56bright natural killer cell, gamma delta T cells, natural killer T cell, regulatory T cell (Treg), and T follicular helper cell were lower in the wild type group. While only several cells including eosinophil, type 17 T helper cell (Th 17), Th 2 cell and CD56dim natural killer cell were higher in the mutated group (Figure 3F). Meanwhile, the activity of some immune functions was downregulated in the KRAS-mutated group, including antigen-presenting cells (APC) co-inhibition, APC co-stimulation, immune checkpoint, cytolytic activity, and HLA molecules (Figure 3G). And then, we did some deeper and more detailed studies. Figure 3H suggested the cytolytic activity score in the KRAS mutant group was lower. The heatmap of HLA gene expression displayed that except for HLA-A, HLA-DQA2, HLADQB2, and HLA-G, most HLA molecules expression was downregulated in the KRAS mutant group (Figure 3I). As for the expression of immune checkpoints, CD40, CD48, CD86, HAVCR2, IDO1, LAIR1, TNFRSF9, and PDCD1LG2 were lower, while the expression of TNFSF15 and NRP1 increased in the KRAS-mutated group (Figure 3J). Overall, these results reflected less immune infiltration, lower APCs activity, lower HLA molecule expression, and lower cytolytic ability in the KRAS-mutated group.

### Biological function analysis of the DEGs

**Figure 4:**
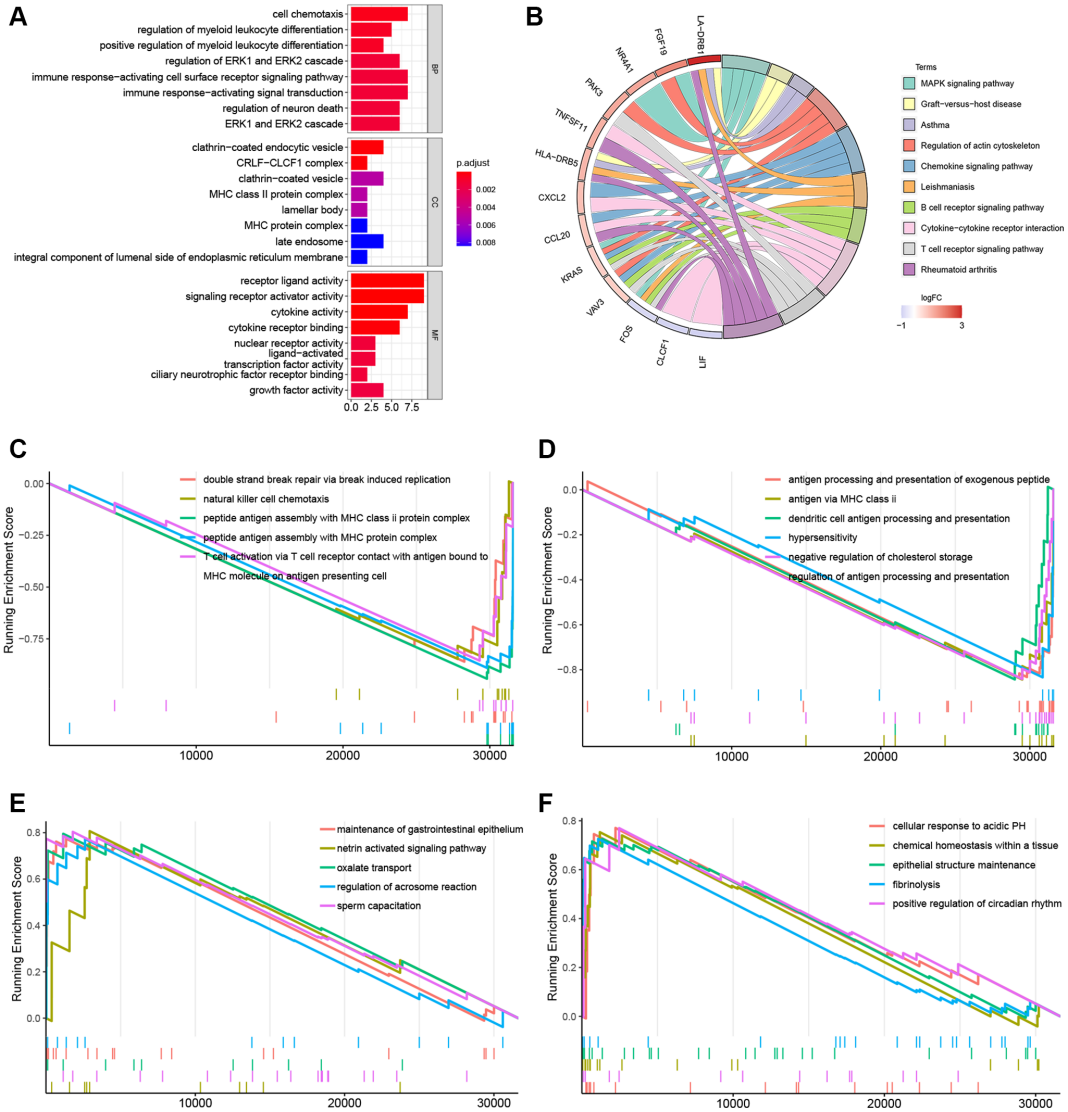
**Gene function enrichment analyses and GSEA plots.** (**A**) Barplot of the GO terms. (**B**) Circle plot of the top 10 KEGG pathways. (**C**, **D**) GSEA plots of the suppressed KEGG pathways in the KRAS-mutated group. (**E**, **F**) GSEA plots of the activated KEGG pathways in the KRAS-mutated group.

Immune-related DEGs including 26 upregulated and 4 downregulated genes were identified between the KRAS-mutated and KRAS-wild groups. To explore underlined molecular mechanisms, GO and KEGG analyses were performed (Figure 4A, 4B). GO terms and the top 10 KEGG pathways revealed that DEGs were involved in many immune-related and cytokine-related biological processes, for instance, cell chemotaxis, regulation of myeloid leukocyte differentiation, immune response-activation cell surface receptor signaling pathway, MHC class II protein complex, MHC protein complex, and cytokine activity receptor ligand activity.

**Figure 5:**
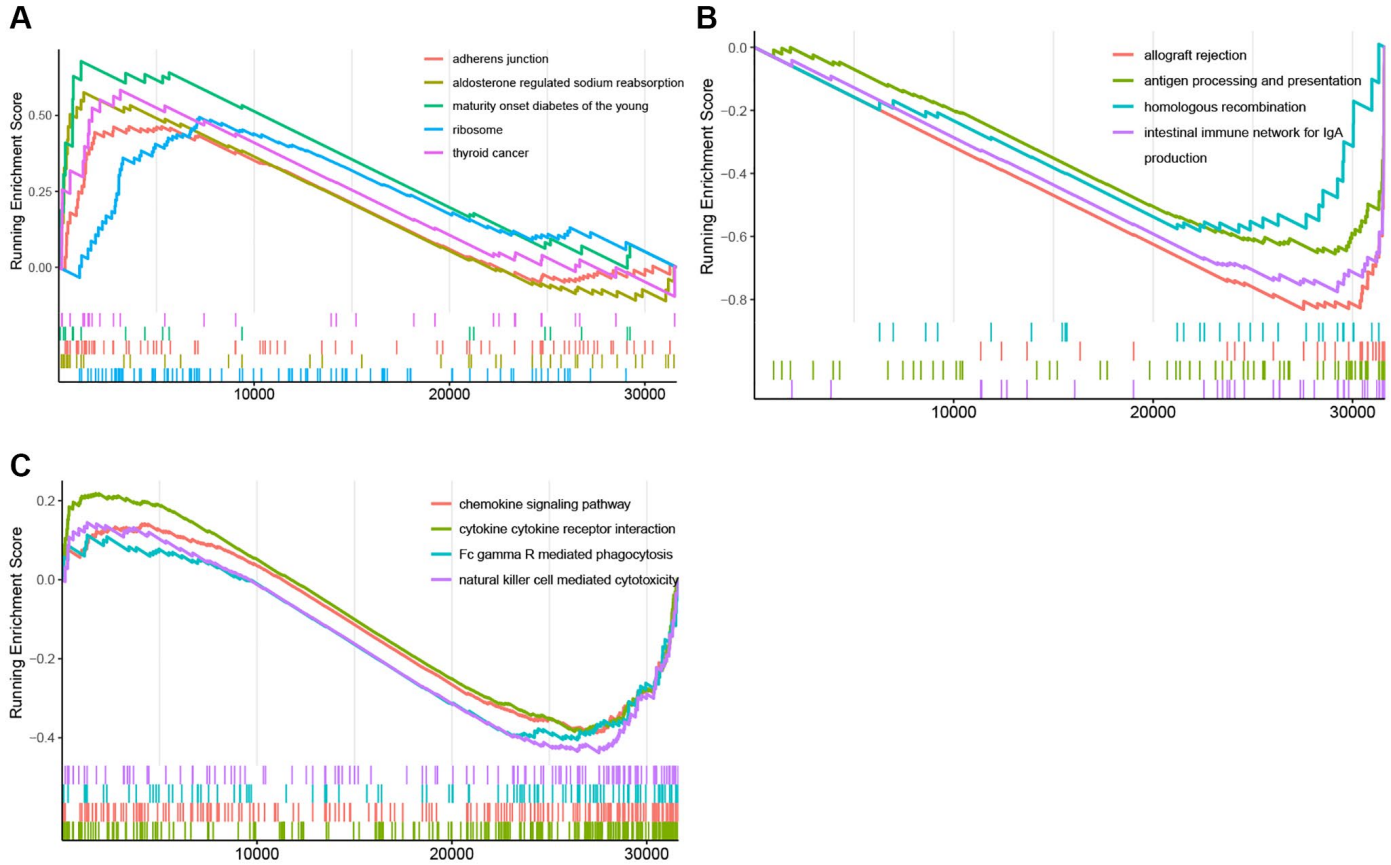
**GSEA plots of Hallmark pathway.** (**A**) GSEA plots of the activated Hallmark pathways in the KRAS-mutated group. (**B**, **C**) GSEA plots of the suppressed Hallmark pathways in the KRAS-mutated group.

In the GSEA plots of KEGG pathways in the KRAS mutant groups, peptide antigen assembly with MHC class I or II protein complex, T cell activation via T cell receptor, and NK cell chemotaxis were inhibited, while netrin activated signaling pathway, oxalated transport, and cellular response to acidic PH were upregulated (Figure 4C–4F). Similarly, in the hallmark pathway GSEA plots, antigen processing and presentation, chemokine signaling pathway, and NK cell mediated cytotoxicity were suppressed, thyroid cancer, ribosome, and adherens junction were activated (Figure 5A–5C). And it reflected the inhibition of antigen processing and presentation, T cell activation and cytokine activity may be the underlying mechanism of the formation of the KRAS special immune landscape.

### Establishment and validation of the gene signature

**Figure 6:**
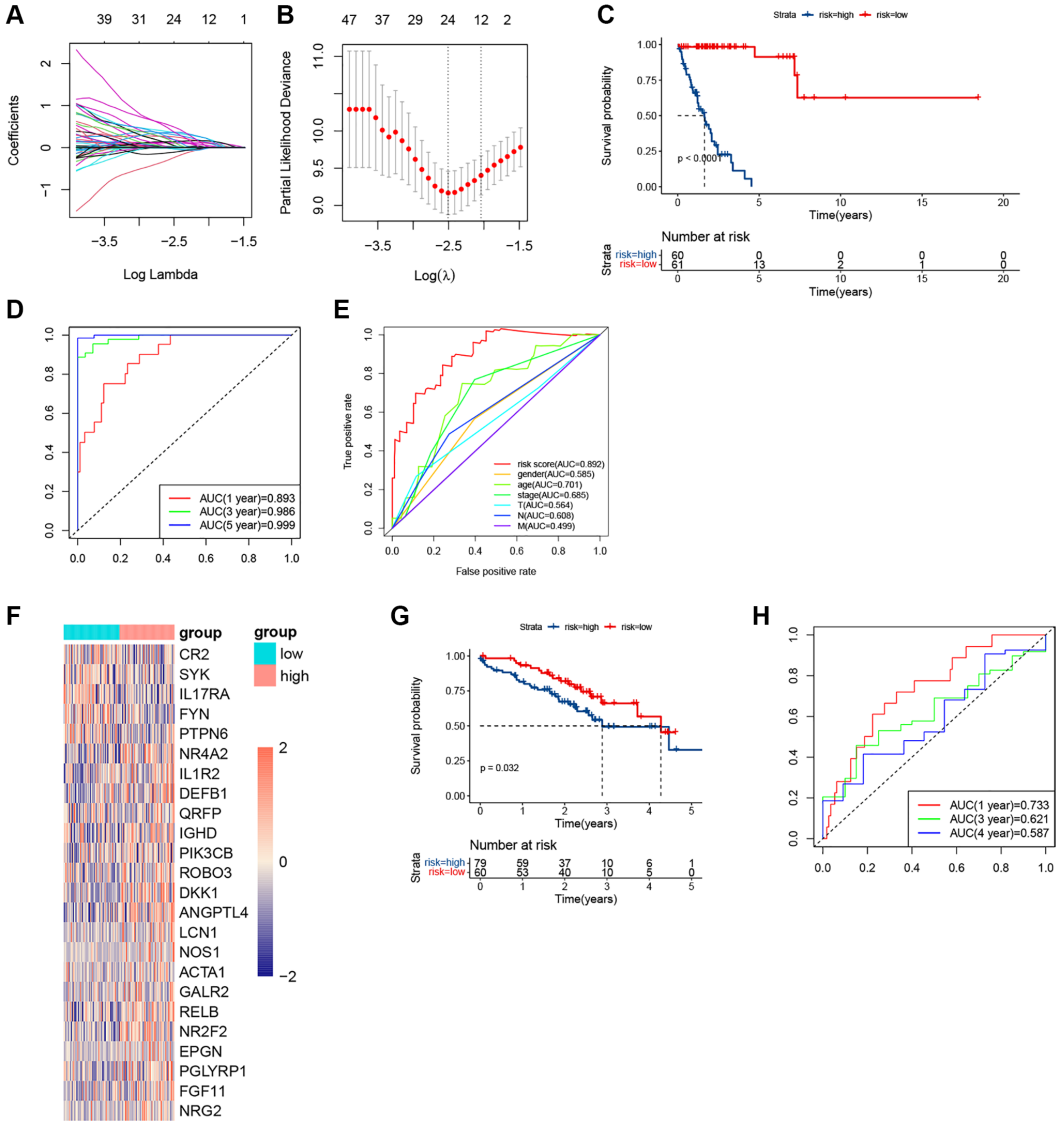
**The plots of LASSO, survival analyses, and AUC in the KRAS-mutated group of training set and validating set.** (**A**, **B**) The plots of LASSO analysis. (**C**) The KM curve in the KRAS-mutated group. (**D**) The time-dependent ROC curves in the KRAS-mutated group. (**E**) The clinicopathologic ROC curves in the KRAS-mutated group. (**F**) The heatmap of the expression values of the prognostic immune-related genes. (**G**) The KM curve in the KRAS-mutated group of validating set. (**H**) The time-dependent ROC curves in the KRAS-mutated group of validating set.

Considering the different immune statuses, we decided to establish an immune-related signature. Among 1700 immune-related genes obtained, 1143 genes were effectively expressed in the TCGA cohort. In the KRAS-mutated group, univariate COX firstly identified 121 genes that were related to the prognosis. And LASSO ultimately identified 24 genes that were used to compose the immune model (Figure 6A, 6B). And samples were assigned to different risk subgroups based on the median value. The low-risk group had a longer OS than the high-risk group (Figure 6C). The AUC for 1, 3, and 5 years were 0.893, 0.986, and 0.999, respectively (Figure 6D). And the AUC of the risk signature was higher than that of all clinical features (Figure 6E). The 24 genes and their coefficient value were shown in Table 2. And Figure 6F was the heatmap of their expression. In the GEO validating set, the immune-related signature also did well in differentiating high- and low-risk subgroups with different prognoses in KRAS-mutated group (*p* = 0.032) (Figure 6G). And in the KRAS-mutated group, the 1-, 3-, and 4-year AUCs were 0.733, 0.621, and 0.587, respectively (Figure 6H). These indicated the signature was stable and performed well in predicting the prognosis of the KRAS-mutated LUAD.

**Table 2:** Gene signature identified by LASSO Cox analysis.

**Gene**	**Full name**	**Coef**
IGHD	Idiopathic growth hormone deficiency	0.023785245
ANGPTL4	Angiopoietin-like 4	0.10973059
DEFB1	Defensin beta 1	0.017366912
PTPN6	Protein tyrosine phosphatase non-receptor type 6	−0.238499217
SYK	Spleen tyrosine kinase	−0.031843183
NR4A2	Nuclear receptor subfamily 4, group A, member 2	0.005309069
RELB	Reticuloendotheliosis viral oncogene homolog B	0.25448851
NR2F2	Nuclear receptor subfamily 2 group F member 2	0.308466697
DKK1	Dickkopf-1	0.10845661
FYN	Fyn	−0.113778857
PIK3CB	Phosphatidylinositol-4,5-bisphosphate 3-kinase catalytic subunit beta	0.046980226
IL17RA	Interleukin 17 Receptor A	−0.032079262
CR2	Complement receptor 2	−0.014593464
IL1R2	Interleukin 1 inhibitory receptor 2	0.00838323
ROBO3	Roundabout 3	0.052143739
EPGN	Epithelial mitogen	0.355319469
ACTA1	Skeletal muscle sarcomeric alpha-actin 1	0.188583774
NOS1	Nitric oxide synthase 1	0.143305847
GALR2	Galanin (GAL) receptor 2	0.190605423
QRFP	Pyroglutamylated RFamide peptide	0.022473031
PGLYRP1	Peptidoglycan recognition protein 1	0.365662324
NRG2	Neuregulin 2	0.493800803
LCN1	Lipocalin 1	0.128260948
FGF11	Fibroblast Growth Factor 11	0.375966194

### Clinicopathological correlation and establishment of the prognostic nomogram

**Figure 7:**
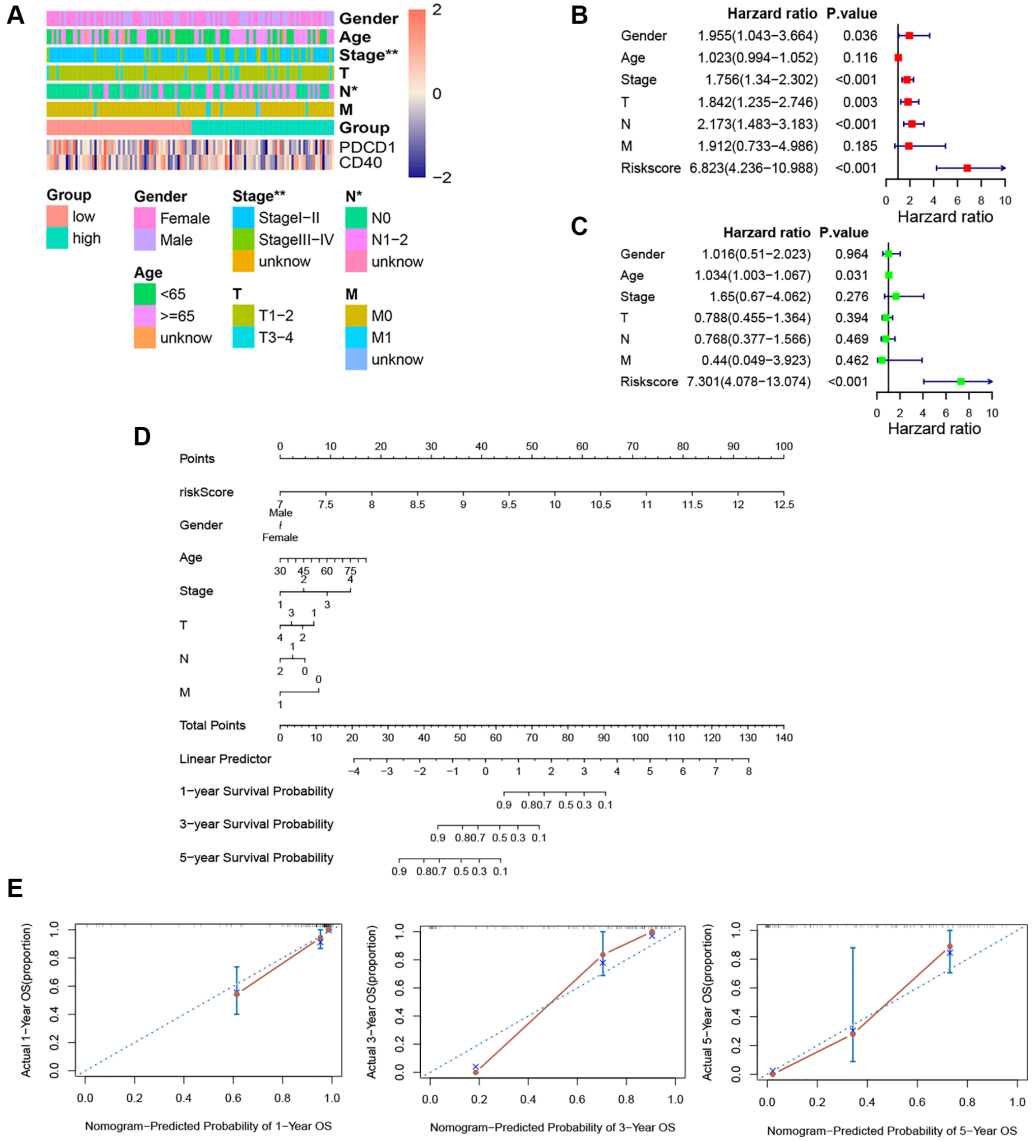
**Independence detection analysis and establishment of the prognostic nomogram.** (**A**) The comparison of clinicopathologic features between high-risk and low-risk groups. (**B**, **C**) The forest plots for univariate and multivariate COX analyses. (**D**) The nomogram based on the multivariate COX analysis. (**E**) Calibration curves for the prediction of 1-, 3- and 5-year overall survival of KRAS-mutated group. (^*^p < 0.05, ^**^p < 0.01, ^***^p < 0.001, ^****^p < 0.0001).

Through chi-analysis, we found that high-risk samples were associated with later stage and more lymph node metastases (Figure 7A). Merged with clinicopathological data, the univariate Cox analysis identified that risk score, gender, stage, and N-stage were potential independent predictive factors (Figure 7B). And according to multivariate Cox result, only the risk score was the independent predictive factor (Figure 7C). The C-index of this nomogram was 0.89 (Figure 7D). And predictive curves of 1-, 3-, and 5-year OS were very close to the curves of real OS (Figure 7E). These elucidated the nomogram had extremely high predictive accuracy.

### Hub genes

**Figure 8:**
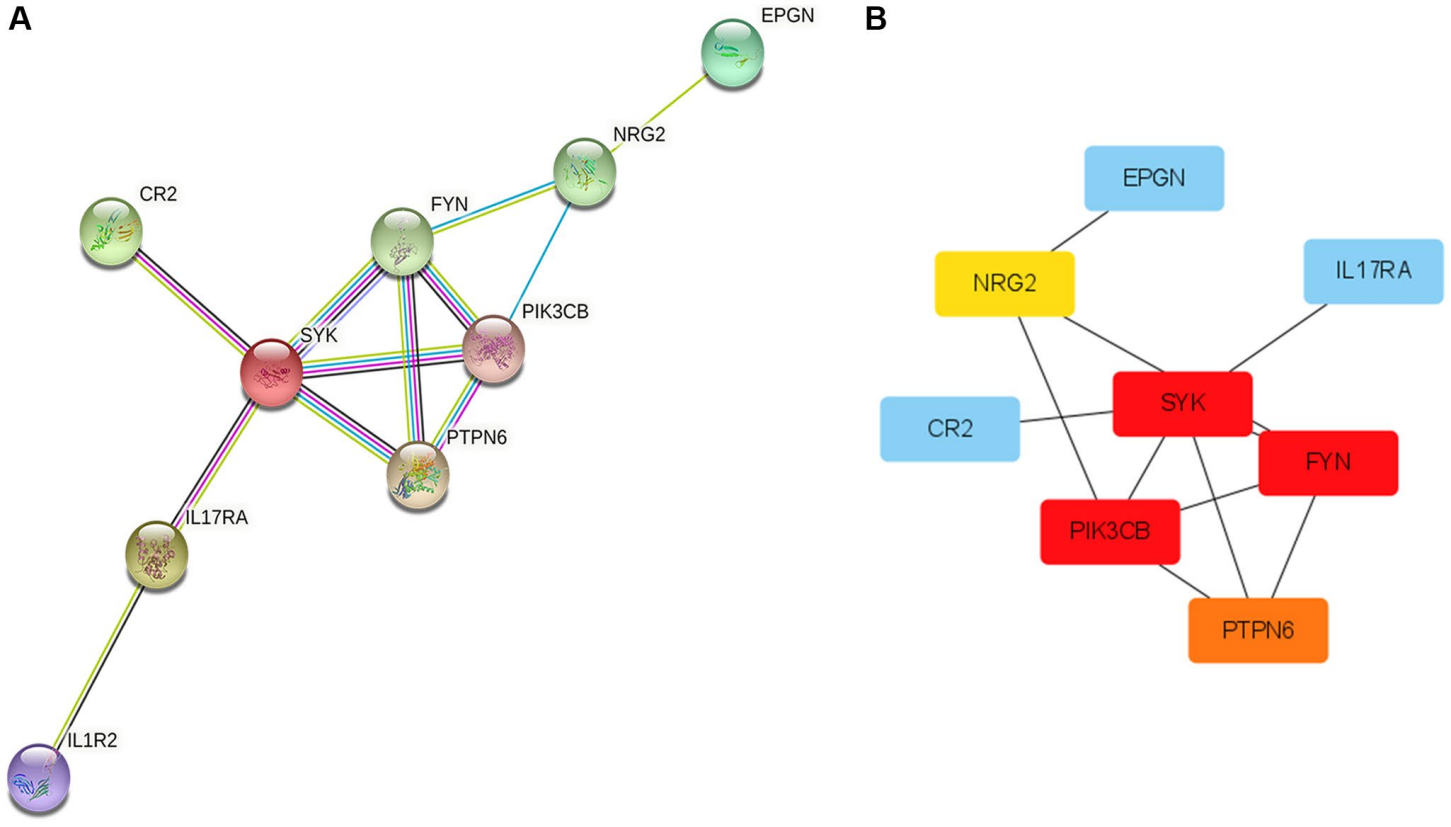
**Protein-Protein Interaction (PPI) network and hub genes.** (**A**) The plot of PPI. Edges represent protein-protein associations, blue edges: known interaction from curated databases, rose red edges: experimentally determined interaction, purple edges: protein homology, green edges: neighborhood genes, black edges: co-expression genes. (**B**) The plot of hub genes.

Protein interaction diagrams were made using the STRING website. Cytoscape was used to screen out the most closely related genes (Figure 8A). The hub gene in the KRAS-mutated type was FYN, SYK, PIK3CB, PTPN6, and NRG2 (Figure 8B).

### Functional enrichment analyses

**Figure 9:**
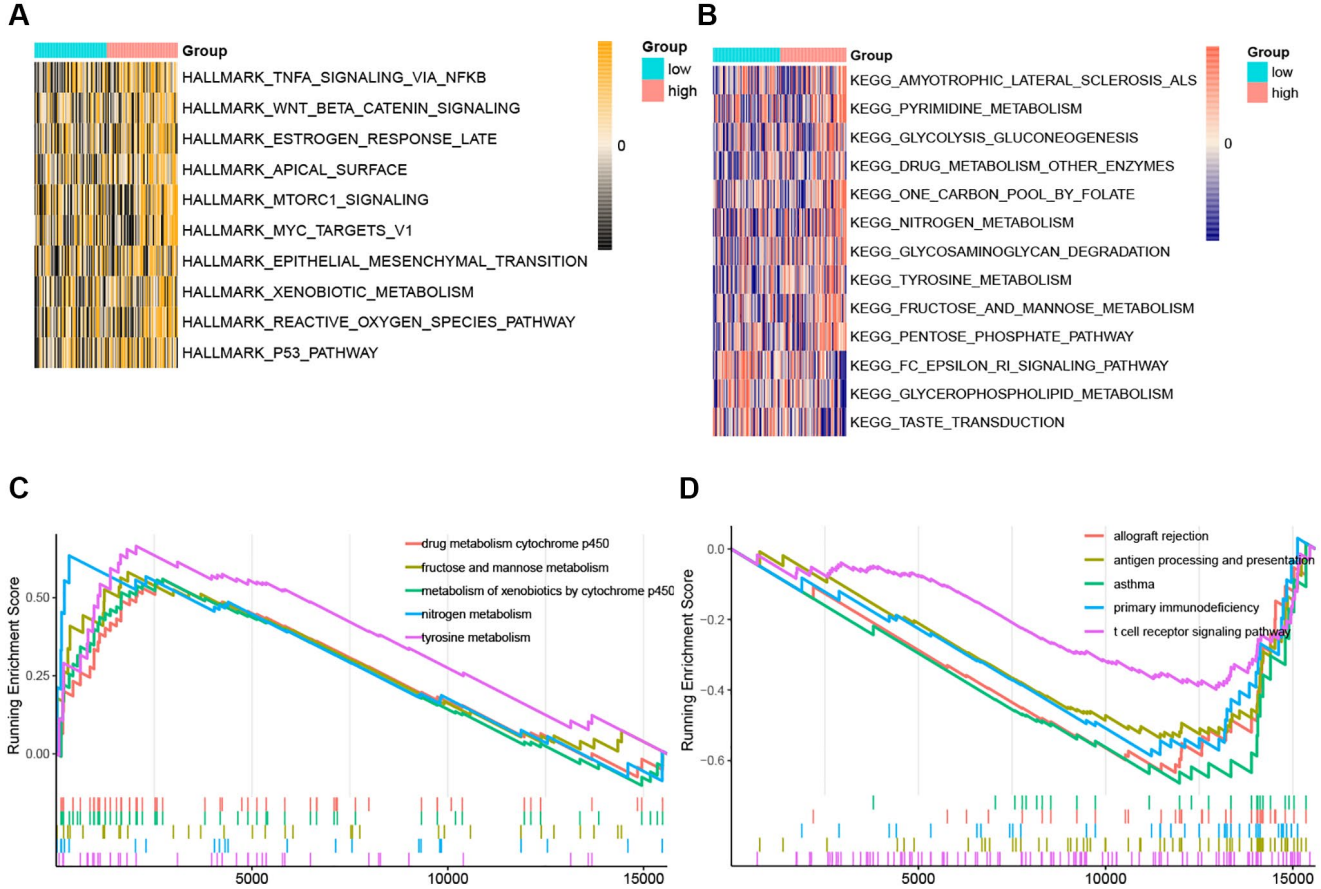
**The ssGSEA score and GSEA of hallmark and KEGG pathways in high-risk and low-risk group.** (**A**) The heatmap of ssGSEA score of hallmark pathways. (**B**) The heatmap of ssGSEA score of KEGG pathways. (**C**) GSEA plots of the activated KEGG pathways in the high-risk group. (**D**) GSEA plots of the suppressed KEGG pathways in the high-risk group.

The Hallmark pathways most correlated with risk scores were displayed in Figure 9A. P53 pathway, reactive oxygen species pathway, xenobiotic metabolism, epithelial-mesenchymal transition, and MYC targets V1 were the top 5 hallmarks pathways positively associated with the risk score. And there was no hallmark pathway significantly negatively related to the risk score. Pentose phosphate pathway, fructose, and mannose metabolism, tyrosine metabolism, glycosaminoglycan degradation, and nitrogen metabolism were the top 5 KEGG pathways positively associated with the risk score (Figure 9B). Fc epsilon RI signaling pathway, glycerophospholipid metabolism, and taste transduction pathway were all KEGG pathways negatively correlated with the risk score (Figure 9B). And the top 5 KEGG pathways with the respectively highest enrichment scores that were activated or inhibited in the high-risk subgroup were shown in the GSEA plots (Figure 9C, 9D). Remarkably, the activity of nitrogen metabolism and tyrosine metabolism was upregulated, while T cell receptor signaling pathway and antigen processing and presentation were suppressed in the high-risk subgroup.

## DISCUSSION

The advent of immunotherapy has greatly extended the survival of many solid tumors, including lung adenocarcinoma. However, the effect of immunotherapy in NSCLC with oncogenic driver alteration was unsatisfactory and immunotherapy should only be recommended after exhaustion of standard chemotherapies and targeted therapies [16]. As a retrospective study for advanced NSCLC receiving immune checkpoint inhibitor monotherapy with at least one oncogenic driver alteration reported, compared with other driver mutations (EGFR, ALK, ROS1), KRAS-mutated patients had the highest ORR to immunotherapy, up to 26% [16]. Hence, it is essential to comprehensively analyze the immune landscape of LUAD patients with KRAS mutation and its underlying mechanisms, making these patients benefit from immunotherapy as much as possible, to prolong the overall survival of the KRAS mutation population.

In our study, we discovered that KRAS mutation was not significantly correlated with patient’s gender, age, T, N, M, and stage. And the frequency of other gene mutations varies between different KRAS status groups. Among them, Skoulidis et al. found the loss of STK11 in the KRAS-mutated LUAD patients promoted immune checkpoint inhibitor resistance [17].

The result of ESTIMATE suggested that the KRAS-mutated group had an inferior stromal score, immune score, and ESTIMATE score, while the purity was superior, which means the KRAS-mutated group may have stronger immune suppression and lower immune infiltration. By our subsequent immune infiltration analysis, the KRAS-mutated group was marked by a low abundance of B cells, CD8+ T cells, dendritic cells (DC), NK cells and macrophages. B cells and CD8+ T cells, the central effector cells in the tumor microenvironment, the low infiltration of them are negatively associated with the prognosis of LUAD [18]. Tumor-associated macrophages (TAMs) usually consist of the M1 type, which inhibits tumor, and the M2 type, which promotes tumor [19]. Therefore, the influence of the increase of macrophages needs to be determined by subsequent analysis of M1 and M2. As for NK cells, they could directly kill the malignant cell in tumor immunosurveillance. And the high infiltration of it was found to be associated with better OS in multiple types of cancers [20]. Moreover, our ssGSEA showed that the cytotoxic activity of killing cells was low, which was related to a higher risk of cancer progression [21]. The deficiency of DC means a decline in the activation, promotion, and maintenance of the anti-tumor immune response [22]. Then, according to the previous studies, tumor-associated neutrophils (TANs) could promote the tumor motility and epithelial-mesenchymal transition (EMT) of LUAD [23, 24].

Notably, the activities of APC co-inhibition, APC co-stimulation, cytolytic activity, and HLA were downregulated in the KRAS-mutated group. As is known to all, the APCs were composed of dendritic cells (DCs), macrophages, and monocytes, which were essential for tissue initiation and facilitation of antitumor response [25]. And according to our result, the abundances of these APCs in the immune environment of KRAS-mutated LUAD were low. Meanwhile, multiple pathways of antigen presentation were suppressed based on our GSEA results. Previous research had demonstrated that the deficiency of antigen processing and presentation is a main immune escape mechanism in cancer [26]. Therefore, the low abundances of important APCs and the loss of antigen processing and presentation in the tumor immune microenvironment might be major reasons for the poor sensitivity to immunotherapy in KRAS-mutated LUAD.

On the other hand, in the KRAS-mutated group, there was fewer abundance of cytotoxic lymphocyte, and the cytolytic activity, as well as the expression of (HLA), were downregulated. Cytotoxic lymphocytes, containing NK cell, NK T cell, and cytotoxic T lymphocyte, take essential roles in killing tumor cells [27]. And the HLA system is a necessary part of the immune system, which could successfully activate cytotoxic lymphocytes to give an effective subsequent immune attack against both pathogen-infected and cancer cells [28]. Thus, the exhaustion of cytotoxic lymphocytes and the dysfunction of cytolytic activity might be another important underlying mechanism for poor immune response in the KRAS-mutated group.

To sum up, the features of the immune landscape in the KRAS-mutated group were low immune cell infiltration, low antigen presentation capacity, and low cytolytic activity. These characteristics can be the breakthrough points to improve the efficacy of immune-related therapy in KRAS-mutated LUAD.

The underlying mechanisms and biological features were further explored by the functional enrichment of the GO and KEGG pathways and GSEA analysis. Based on these results, we can know that the function of DEGs between KRAS-mutated and KRAS-wild groups mainly enriched on MHC molecular, antigen processing and presentation, immune response-activating cells surface receptor signaling pathway, ERK1, and ERK2 cascade, and cytokine-related pathway. Moreover, the outcomes of GSEA further suggested that both the assembly of MHC class I molecules with endogenous antigens and the assembly of MHC class II molecules with exogenous antigens were downregulated in the KRAS-mutated groups. On the other hand, T cell activation by contacting T cell receptor (TCR) with antigen bound to MHC molecules on APC was also suppressed. These were all the underlying mechanisms of the dysfunction of cytolytic activity and the decline of immune response in KRAS-mutated LUAD samples. This suggested that KRAS-mutated patients may benefit from adoptive cellular immunotherapy, including tumor-infiltrating lymphocytes (TIL) therapy and chimeric antigen receptor-modified T cells (CAR-T cells) therapy. To date, several experimental studies have made some related attempts. Srivastava et al. [29] reported, in Oxaliplatin pretreated KRAS-mutated lung cancer mouse, chimeric antigen receptor-modified T cells (CAR-T cells) therapy increased cancer sensitivity to anti-PD-L1 therapy. Tran et al. [30] discovered that the infusion of CD8+ T cells targeting KRAS G12D mutation colorectal cancer patients presented a good effect of antitumor immunotherapy.

As for the ERK1 and ERK2 cascade, it is essential for cells to regulate fundamental cell functions, including cell cycle progression, survival, cell migration, and differentiation [31]. And it participated in the RAS/RAF/MEK/ERK (MAPK) pathway, which is keenly correlated to tumorigenesis [32]. Previous studies had demonstrated that RAS mutation could result in permanent activation of this process. Compared with NRAS and HRAS, KRAS mutation has a stronger ability to activate RAS/RAF/MEK/ERK signaling. Hence, some of our outcomes are consistent with previous research, increasing the credibility of our study. Survival time predictive values were similar to the corresponding real survival time.

The prediction ability of our immune-related signature is outstanding. Both AUC of the signature and C-index of the corresponding nomogram were extremely high. Therefore, it may have the value of application and promotion in the clinic. The hub gene in the KRAS-mutated type was FYN, SYK, PIK3CB, PTPN6, and NRG2. FYN, whose protein belongs to tyrosine-protein kinase, takes part in the development and activation of T lymphocytes. The overexpressed FYN had been demonstrated to be associated with a good prognosis of LUAD, suppressing the EMT via down-regulating the PI3K/AKT pathway [33]. Spleen tyrosine kinase (SYK) takes an important role in angiogenesis, progression, and metastasis of lung cancer [34]. The decreased expression of SYK was inversely correlated with the survival of non-small-cell lung cancer [35]. Tyrosine-protein phosphatase non-receptor type 6 (PTPN6), also called protein tyrosine phosphatase-1 (SHP-1), was discovered to serve as an anti-tumor gene in lung cancer [36]. PIK3CB is the gene encoding PI3Kp110β, which is one of the functional isoforms of PI3k. By promoting tumor growth, high PIK3CB expression was related to the worse prognosis of LUAD [37]. Neuregulin 2 (NRG2) is one of the growth and differentiation factors related to the epidermal growth factor [38]. It has been demonstrated that NSCLC with NRG1 and NRG2 fusions is more aggressive and has stronger drug resistance. Zhao et al. [39] discovered that NRG2 could promote the migration of human glioma. However, the role of NRG2 in lung cancer was still unclear. The antitumor functions of FYN, SYK, and PTPN6 in lung cancer have been validated by previous research, which was consistent with our study. But there are no direct reports of NRG2 on LUAD yet. Our study was the first to suggest that NRG2 could be a new biomarker in the prognosis of LUAD and it needs to be researched for subsequent study.

Undeniably, our article still needs some improvement. Firstly, our research was based on a public database and bioinformatic technology, further laboratory and clinical studies are needed to verify our findings. Secondly, our research only analyzed LUAD sequencing data, in the future, we could comprehensively analyze the immune landscape characteristics of KRAS-mutated LUAD from multiple perspectives, such as proteomics and metabolomics.

## CONCLUSION

Taken together, the immune landscape of KRAS-mutated LUAD was featured with the exhaustion of APC, the low abundance of cytotoxic lymphocytes, the dysfunction of and cytolytic activity, and the loss of antigen processing and presentation. Moreover, the assembly of MHC class I and MHC class II molecules with antigens was downregulated, and T cell activation by contacting T cell receptor (TCR) with antigen bound to MHC molecules on APC was also suppressed. These were important underlying mechanisms in the formation of KRAS-mutated immune microenvironment. Our study elucidated the special immune landscape characteristics of LUAD with KRAS mutation, and offered a novel insight to improving the immunotherapy response in KRAS-mutated LUAD patients and established a prognostic gene signature with high accuracy.
